# Perspectives on Colistin—An Old Antibiotic with Renewed Relevance in Modern Medicine

**DOI:** 10.3390/ijms27073115

**Published:** 2026-03-30

**Authors:** Wioleta Lewandowska, Izabela Swiecicka

**Affiliations:** 1 Doctoral School, University of Bialystok, 1K Ciołkowski Street, 15-245 Bialystok, Poland; w.lewandowska@uwb.edu.pl; 2 Department of Microbiology and Biotechnology, Faculty of Biology, University of Bialystok, 1J Ciołkowski Street, 15-245 Bialystok, Poland; 3 Laboratory of Applied Microbiology, Faculty of Biology, University of Bialystok, 1J Ciołkowski Street, 15-245 Bialystok, Poland

**Keywords:** colistin, polymyxin, combination therapy, susceptibility testing

## Abstract

Antibiotic resistance among bacteria represents a major challenge in modern medicine. The absence of antibiotics effective against multidrug-resistant pathogens triggers interest in reviving older antibiotics. This review aims to provide a focused and updated perspective on the reintroduction of polymyxin antibiotics, with a particular emphasis on colistin, a cyclic oligopeptide initially used in the 1950s and 1960s. We analyze colistin from multiple perspectives, including (i) its historical and contemporary clinical applications, (ii) pharmacokinetic and pharmacodynamic properties, and (iii) use in veterinary medicine and animal husbandry. Key unresolved issues are highlighted, such as colistin toxicity, challenges in susceptibility testing, the emergence of resistance, including the *mcr* gene variants, and inconsistent clinical evidence supporting combination therapy. By integrating historical background with current data, this review provides a comprehensive overview of the therapeutic relevance, limitations, and ongoing challenges associated with colistin in the era of multidrug-resistant Gram-negative infections.

## 1. Introduction

The polymyxin class of antibiotics, comprising five structurally and chemically distinct non-ribosomal cyclic oligopeptides (A, B, C, D, and E), is represented in current clinical practice mostly by polymyxin B and polymyxin E, also known as colistin [1]. These antibiotics differ structurally by only a single amino-acid substitution and share a common amphipathic structure composed of a cyclic peptide ring, a linear tripeptide chain, and a hydrophobic fatty acyl tail. At physiological pH, polymyxins are strongly polycationic due to the presence of five L-α,γ-diaminobutyric acid (Dab) residues. This amphipathic nature underlies their ability to interact with bacterial membranes but also contributes to non-specific adsorption to various surfaces [2]. The polymyxins now play a critical role primarily against Gram-negative bacteria that cause severe infections. The rapid global spread of multidrug-resistant Gram-negative pathogens has revived clinical interest in older antimicrobial agents, positioning colistin as a crucial last-resort therapy for infections caused by carbapenem-resistant bacteria. However, despite its clinical importance, the use of colistin remains associated with numerous challenges, including difficulties with susceptibility testing, complex pharmacokinetics, significant toxicity, and the emergence of plasmid-mediated resistance mechanisms. This review focuses on colistin, given its status as the most frequently administered antibiotic within the polymyxin class [3].

**Figure 1:**
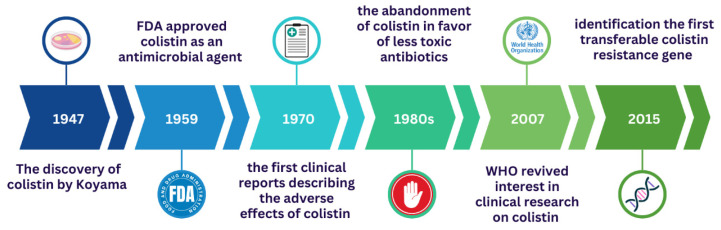
Timeline showing the discovery of colistin and its implementation in the clinic. Figure created using Canva (https://www.canva.com (accessed on 12 March 2026), Canva Pty Ltd., Sydney, Australia).

The discovery of colistin by Koyama in 1947 sparked renewed optimism for transforming the trajectory of human medicine, particularly in the treatment of life-threatening bacterial infections. Koyama reported colistin as a secondary metabolite of the Gram-positive soil bacterium *Paenibacillus polymyxa* subsp. *colistinus* [4]. Colistin was commercialized and used for the first time in the 1950s as an intravenous formulation. In 1959, the U.S. Food and Drug Administration (FDA) approved colistin as an antimicrobial agent against Gram-negative bacteria (Figure 1), due to its bactericidal activity for the treatment of various types of infectious diseases, e.g., diarrhea, urinary tract infections, eye, and ear infections [1]. In fact, colistin was one of the first antibiotics with significant activity against Gram-negative bacteria, notably *Pseudomonas aeruginosa*, *Acinetobacter baumannii*, and *Klebsiella pneumoniae* [5], but also against *E. coli* [6] and other *Enterobacteriaceae* [1].

The escalating and global problem of antibiotic resistance among bacteria badly necessitates the development of strategies and tools to combat this critically important medical challenge. It is estimated that 39 million deaths will be attributable to antibiotic resistance by 2050 [7]. In 2023, approximately one in six laboratory-confirmed infections worldwide were caused by bacteria resistant to antibiotics [7]. Analysis of the extent of resistance in 93 infection type–pathogen–antibiotic combinations indicates a global pattern of antibiotic resistance characterized by widespread resistance to essential first-, second-, and last-resort antibiotics, but with substantial variation among pathogens and regions [7]. The absence of new antibiotics effective against multidrug-resistant pathogens, largely due to the stagnation of the antibiotic discovery pipeline, has led to renewed interest in reviving older antibiotics, particularly the polymyxins.

As early as 1970, the first clinical reports describing the adverse effects of colistin emerged, signaling the collapse of the concept of an optimally effective antibiotic for combating Gram-negative bacterial infections [8]. Colistin was subsequently abandoned in the 1980s in favor of other, less toxic broad-spectrum antibiotics [9]. Nevertheless, despite its adverse effects being well established, the resurgence of multidrug-resistant Gram-negative bacteria led to the reinstatement of colistin in clinical therapy. Colistin regained significance in antimicrobial therapeutics during the early 2000s [9]. A few years later, in 2005, Falagas and Kasiakou [6] highlighted a rising trend toward the reintroduction of colistin in the management of severe, multidrug-resistant bacterial infections. In response to the escalating global problem of antimicrobial resistance, in 2007, the World Health Organization (WHO) revived interest in clinical research on this antibiotic (Figure 1) and conducted a reclassification of the polymyxin class as a major agent for the treatment of infections caused by multidrug-resistant Gram-negative bacteria, e.g., nosocomial infections, ventilator-associated pneumonia, urinary tract infections [10,11,12]. Further investigations were conducted to understand the molecular and cellular mechanisms of colistin resistance. The intensive development of analytical and molecular techniques allowed the identification of the first transferable colistin the *mcr-1* resistance gene (a mobile colistin resistance gene), which took place in 2015 [13]. Since the initial discovery of the plasmid-mediated colistin resistance *mcr-1* gene, several additional variants (from *mcr-2* to *mcr-10*) have been identified in diverse bacterial species isolated from clinical, animal, and environmental sources [14,15]. For many years, colistin has been extensively used in veterinary practice both as a growth promoter and for the prevention and treatment of bacterial infections in livestock [16]. Currently, colistin is used as a last-resort treatment against multidrug-resistant (MDR) and extensively drug-resistant (XDR) Gram-negative bacteria [1]. Given that the bacterial mechanisms underlying colistin resistance have been comprehensively characterized [17,18,19], this review focuses on other issues pertaining to colistin administration, the relevance of this antibiotic in modern medicine, and current perspectives in the treatment of severe bacterial infections. Review highlights several important limitations associated with the reintroduction of colistin into contemporary clinical practice. In particular, we discuss the challenges related to reliable colistin susceptibility testing and the optimization of dosing strategies in patients. The clinical use of colistin remains challenging due to its complex pharmacokinetic profile, narrow therapeutic window, difficulties in standardized susceptibility testing, and the significant risk of nephrotoxicity associated with systemic administration. We specifically address these challenging aspects of colistin therapy and emphasize that the anticipated therapeutic benefits of colistin-based combination regimens are not consistently supported by current experimental and clinical evidence. Rather than providing a purely descriptive overview, this review focuses on critically integrating molecular, pharmacological, and clinical evidence to assess the actual therapeutic relevance of colistin in modern medicine. Review addresses a central question: does the renewed clinical use of colistin provide a tangible therapeutic advantage, or does its application remain constrained by pharmacological limitations, methodological challenges, and inconsistent clinical evidence?

For this review, relevant articles were identified through a comprehensive search of the PubMed database. The literature surveyed spans from the initial discovery of colistin to the present, with particular emphasis on studies published in the last two decades. The search strategy included combinations of the following terms: ‘colistin’, ‘polymyxin’, ‘Gram-negative bacteria’, ‘colistin therapy’, and ‘colistin combination therapy’. Original research articles, clinical studies, and relevant review papers addressing the pharmacology, clinical use, and resistance mechanisms associated with colistin were considered.

## 2. Mechanism of Colistin Action

**Figure 2:**
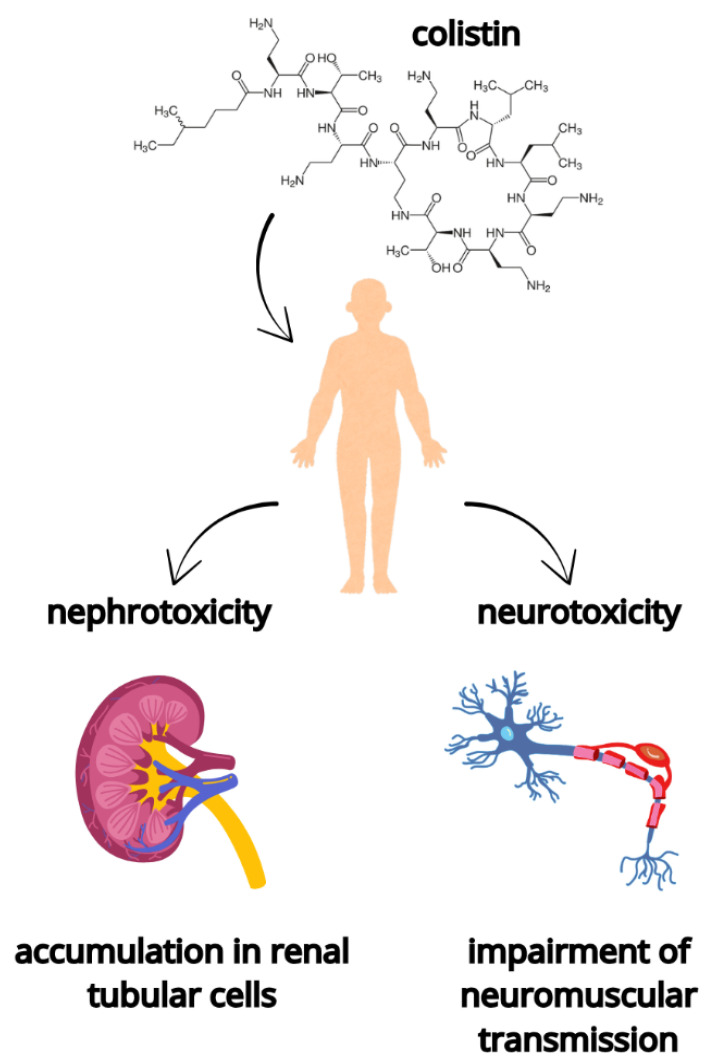
The two most commonly observed adverse effects associated with colistin administration involving renal and nervous system dysfunction. Figure created using Canva (Canva Pty Ltd., Sydney, Australia).

Colistin is a cyclic oligopeptide antibiotic that affects bacterial cells by binding to the lipopolysaccharide (LPS) of the outer cell membranes via electrostatic interactions. This binding disrupts the outer membrane structure, leading to its disorganization and subsequent alteration [20]. Colistin is composed of a decapeptide structure containing five L-α,γ-diaminobutyric acid residues and an N-terminal fatty acyl chain, which together confer a strongly amphipathic character essential for interaction with the lipopolysaccharide layer (Figure 2) [21]. The initial reaction of colistin with the bacterial membrane occurs via electrostatic interactions between the cationic diaminobutyric acid (Dab) residues of colistin and anionic phosphate groups on the lipid A moiety of LPS in the outer membrane of the Gram-negative bacteria. Then, colistin competitively displaces the divalent cations Mg^2+^ and Ca^2+^ from the negatively charged phosphate groups of membrane lipids, destabilizing the LPS molecules, therefore allowing for the cellular uptake of colistin. Colistin attaches and binds to the lipid A component of LPS, resulting in the derangement of the outer membrane [1,10,22]. Moreover, the binding of colistin to the lipid A component of the LPS molecule results in a significant reduction in LPS endotoxic activity. Additionally, colistin inhibits LPS-induced leukocyte activation, leading to a decrease in interleukin production [23].

Two forms of colistin are clinically available for human treatment: colistin sulfate and colistin methanesulfonate (CMS), also known as colistimethate sodium (Table 1) [20]. CMS is produced by the reaction of colistin with formaldehyde and sodium bisulfite, which leads to the addition of a sulfomethyl group to the primary amines of colistin [24]. Only a fraction of CMS, considered as a prodrug, is hydrolyzed into active colistin [25], whereas most of the dose is eliminated via renal excretion [23]. CMS acts as a prodrug because in aqueous solutions it undergoes hydrolysis to form a complex mixture of partially sulfomethylated derivatives and colistin [24]. Colistin sulphate is the only form of colistin approved for use in pig production for the control of intestinal infections caused by *Enterobacteriaceae*, in particular by *E. coli* and *Salmonella* [1]. The penetration of colistin through the blood–brain barrier is minimal [26], but data on colistin penetration into pulmonary tissue remain inconsistent. Clinical experience demonstrates the efficacy of the treatment of pneumonia via both intravenous and inhalation routes of administration. Orally administered colistin exhibits limited absorption, while inhalation delivery allows for variable uptake influenced by lung pathology and the nebulization system used [23].

## 3. Current Approaches and Standards in MIC Determination

In 2016, the WHO classified polymyxins into the group of critically important antimicrobials with the highest priority for human medicine [27]. Whereas the World Organization for Animal Health (WOAH, formerly OIE) classified polymyxins as veterinary antimicrobial agents of high importance [28]. In 2019, the European Medicine Agency (EMA) updated its 2014 advice on the categorization of antibiotics used in veterinary medicine, which could pose a risk to human public health. Polymyxins are classified into category B, called ‘restricted’, which includes antimicrobials from the WHO’s highest priority list, and should only be used in food-producing and companion animals for the treatment of infections when there is no alternative antibiotic from category C or D [29].

Antimicrobial susceptibility testing for colistin is critical in the era of rising multidrug-resistant Gram-negative infections. Reliable determination of colistin susceptibility not only informs appropriate therapeutic decisions but is also essential for monitoring the emergence and spread of resistance. Colistin exhibits a strong tendency to adsorb to negatively charged surfaces, including plastics commonly used in laboratory equipment such as polystyrene. This interaction results from electrostatic attraction between the positively charged regions of the colistin molecule and the negatively charged surface of the plastic material, potentially reducing the effective antibiotic concentration in experimental systems [30]. Assessment of the colistin minimum inhibitory concentration (MIC) remains challenging, as the results are significantly influenced by the methods used for susceptibility testing and the type of bacterial media used in dilution assays [31]. According to current recommendations, the standardized method is broth microdilution using cation-adjusted Mueller-Hinton broth, with sulfate salts of colistin in plain polystyrene trays without additives such as polysorbate-80 [32,33]. Interpretive breakpoints for colistin susceptibility are currently available from the European Committee on Antimicrobial Susceptibility Testing (EUCAST) and the Clinical Laboratory Standards Institute (CLSI) (Table 2). Both EUCAST and CLSI recommend that susceptibility testing be performed exclusively with the active form, colistin sulfate, rather than with its inactive prodrug, colistin methanesulfonate (CMS) [34]. Agar dilution, disk diffusion, and gradient diffusion are not currently recommended by CLSI and EUCAST as these methods yield unacceptably high error rates compared to broth microdilution [33].

Testing for susceptibility to colistin is inherently associated with heteroresistance, defined as the presence of distinct bacterial subpopulations displaying different levels of susceptibility within a single isolate [35]. Population analysis profiles (PAPs), performed during time–kill or dynamic pharmacokinetic/pharmacodynamic experiments, have demonstrated that such resistant subpopulations may exist even when isolates are classified as colistin-susceptible based on MIC values. Studies on *Pseudomonas aeruginosa* using dynamic in vitro pharmacokinetic models showed comparable bactericidal activity across different dosing intervals [36]. Similar heteroresistance has been reported in clinical isolates of *Acinetobacter baumannii*, in which small resistant subpopulations were detected despite overall susceptibility by standard MIC testing [37]. These findings highlight that conventional susceptibility testing may underestimate the presence of resistant subpopulations during colistin therapy.

**Table 2:** CLSI and EUCAST breakpoints for susceptibility testing of colistin [38,39].

Minimum Inhibitory Concentrations [mg/L]						
	*A. baumanii*		*P. aeruginosa*		*Enterobacteriaceae*	
	S	R	S	R	S	R
CLSI	≤2	≥4	≤2	≥8	-	-
EUCAST	≤2	>2	≤4	>4	≤2	>2

S, susceptible; R, resistant; CLSI, the Clinical & Laboratory Standards Institute; EUCAST, the European Committee on Antimicrobial Susceptibility Testing.

## 4. Pharmacokinetics (PK) and Pharmacodynamics (PD), Colistin Dosage

Over the last decade, numerous studies have been conducted to generate the pharmacokinetic (PK) and pharmacodynamic (PD) properties of colistin and the relationship between PK and PD to obtain the pharmacological information needed to guide colistin’s optimal use in patients [40]. The murine thigh infection model is widely used to assess in vivo antimicrobial efficacy and has demonstrated good translational relevance to human infections. Furthermore, the thigh infection model is considered a suitable preclinical simulator, as it enables the systemic dissemination of bacteria into the bloodstream and internal organs, resembling the clinical manifestations of bacteremia [41]. The pharmacokinetic/pharmacodynamic index (PK/PD) of colistin is characterized by the ratio of the area under the unbound concentration–time curve to the MIC (ƒAUC/MIC). This index demonstrates the highest predictive value for bacterial killing by colistin [40]. The minimum inhibitory concentration (MIC) is a relevant PD parameter. Consideration of MIC in conjunction with other PK parameters helps in determining the proper use of antibiotics.

Modern models and simulations integrating PK and PD are being employed to predict plasma concentrations and suggest a suitable treatment scheme. Typically, a range of doses is tested, and the optimal dose is selected based on the pharmacokinetic profile that yields the best results [42]. Optimal dosage regimens for colistin have been investigated over time based on a population pharmacokinetic model and the pharmacokinetic/pharmacodynamic (PK/PD) index of colistin [41].

In clinical practice, colistin is most administered as its inactive prodrug, colistin methanesulfonate (CMS). To achieve the desired therapeutic effect, an appropriate dose of CMS must be administered to reach a target plasma concentration of approximately 2 mg/L. Maintaining this target concentration is crucial, as colistin exhibits a narrow therapeutic window. Therapeutic drug monitoring studies suggest that a target steady-state average total plasma concentration (Css, avg) of approximately 2 mg/L provides an optimal balance between antibacterial efficacy and toxicity. However, plasma concentrations exceeding ~2–2.5 mg/L have been associated with an increased risk of nephrotoxicity [42,43]. Pharmacokinetics of colistin following colistin methanesulfonate administration vary substantially across patient populations. In critically ill patients, considerable individual variability has been reported, with renal function representing the main determinant of drug exposure. Impaired renal clearance reduces CMS elimination and increases the fraction of colistin converted to the active form, leading to higher plasma concentrations. Consequently, CMS dosing must be adjusted according to renal function, and loading doses are often recommended to achieve therapeutic concentrations more rapidly [44]. In patients undergoing haemodialysis or continuous renal replacement therapy, both CMS and colistin may be removed by extracorporeal circulation, requiring dose modification and, in some cases, supplemental dosing after dialysis sessions. In contrast, patients with cystic fibrosis may exhibit lower systemic exposure to colistin, suggesting increased drug clearance, whereas burn patients generally display pharmacokinetic profiles comparable to those of other critically ill individuals. Overall, the marked variability in colistin pharmacokinetics highlights the importance of individualized dosing strategies and careful clinical monitoring [44].

Among the adverse reactions linked to colistin administration, renal toxicity is the most frequently reported (Figure 2), as the drug is primarily eliminated by the kidneys [45]. Several factors have been identified as potentiating the nephrotoxic effects of colistin in critically ill patients, including sepsis, shock, and hypoalbuminemia [46]. Age, disease status, duration, dose, and concomitant administration of other potentially nephrotoxic drugs, e.g., vancomycin and nonsteroidal anti-inflammatory drugs, are additional risk factors for nephrotoxicity. Patients with a high body mass index may be at an increased risk of colistin-associated nephrotoxicity, which may be a consequence of dosing according to actual body weight [47]. Renal dysfunction frequently occurs in patients receiving CMS, necessitating close monitoring of kidney function. In many cases, renal impairment is reversible after discontinuation of CMS, although the severity may vary among patients [23]. Compared with earlier experiences with CMS, current clinical practice benefits from improved renal monitoring, enhanced management strategies for kidney dysfunction, and heightened awareness of the additional risk associated with concomitant use of other potentially nephrotoxic agents.

The second most frequently observed adverse effect of colistin administration involves its interaction with neurons (Figure 2). The most severe neurotoxic event is neuromuscular blockade, which may manifest as a myasthenia-like syndrome or as respiratory muscle paralysis resulting in apnea [46]. Neurotoxicity is also dose-dependent and may be triggered by the presence of risk factors like hypoxia, co-administration of muscle relaxants, sedatives, or steroids [48]. Neurotoxicity has been reported less frequently in the literature compared with nephrotoxicity. However, symptoms associated with neurotoxicity might be missed due to the lack of objective criteria. Current investigations focus on critically ill, sedated, or unresponsive patients, which may result in underestimation of colistin-associated neurotoxicity [49]. Overall, the clinical use of colistin requires careful dose optimization and close monitoring due to its narrow therapeutic window and the risk of dose-dependent toxicity.

## 5. Future Perspective—Combination Therapy

A key question is whether the mechanistic rationale for colistin-based combination therapy translates into consistent and clinically meaningful outcomes. The available evidence can be broadly categorized into in vitro studies, animal models, and clinical investigations, each providing a different level of evidence and translational relevance. Despite extensive investigation, the outcomes of these combination regimens remain controversial. Currently, the reintroduction of colistin is accompanied by combination therapy to enhance efficacy and safety [41,50,51,52,53,54]. The available evidence encompasses both in vitro studies evaluating the direct effects of antimicrobial combinations on bacterial pathogens and in vivo investigations assessing the clinical outcomes of such therapeutic strategies in patients. However, the outcomes of these combination regimens remain controversial.

In vitro investigations indicate that combination therapies may enhance the antibacterial activity of colistin [55]. Research conducted in vitro on biofilm-forming pathogens suggests that combining colistin with other antimicrobial agents may yield intriguing and potentially beneficial results. Copur and co-investigators [56] reported synergistic effects for the combinations of meropenem/colistin and meropenem/ciprofloxacin, as demonstrated by checkerboard and time–kill assays. Taşkın Kafa and Hasbek [57] reported that the percentage of biofilm inhibition was 30.8% when colistin was used alone, while the biofilm inhibition rates of colistin/meropenem and colistin/ciprofloxacin were 92.4% and 91.7%, respectively. The combination of doripenem and colistin resulted in enhanced killing of *Pseudomonas aeruginosa* biofilm-embedded cells by colistin in both carbapenem-susceptible and carbapenem-resistant strains, and the combination minimized the emergence of colistin resistance [58]. Gómez-Junyent et al. [59] reported that combinations of colistin plus ceftolozane/tazobactam and meropenem were the most appropriate treatments for biofilm-related infections caused by XDR and MDR *P. aeruginosa* strains, respectively, in an in vitro biofilm pharmacodynamic model. Collectively, these studies suggest that colistin-based combinations may enhance antibacterial activity against biofilm-associated infections. Despite these promising in vitro findings, their translation into clinical efficacy remains uncertain. Collectively, these studies suggest that colistin-based combinations may enhance antibacterial activity against biofilm-associated infections; however, their translation into clinical efficacy remains uncertain.

Although combination therapies are often proposed to enhance the antibacterial efficacy of colistin, current evidence indicates that their clinical benefits remain inconsistent and often fail to translate into improved patient outcomes (Table 3). The use of colistin combination therapy is considered in two clinical contexts: (i) infections caused by carbapenem-resistant strains, and (ii) infections caused by biofilm-forming pathogens. Multiple antibiotic combinations have been suggested, such as colistin combinations with carbapenems, tigecycline, rifampicin, sulbactam, ampicillin/sulbactam, and ceftolozan/tazobactam (Table 3). Clinical studies have reported potential benefits of combination therapy, including reduced all-cause mortality and improved microbiological response [60]. However, these findings are largely derived from observational studies and remain inconsistent. The therapeutic outcomes of combination therapy show considerable variability (Table 3). This is linked to notable methodological limitations, which might be influenced by unmeasured factors that could significantly affect the link between the administered antimicrobials and the observed outcomes. Intrinsic limitations associated with the retrospective study design, such as the risk of selection and information biases and the inherently lower level of evidence, further restrict the external validity of the study results [60]. The limitations of this type of study include the fact that the severity of illness, the timing of antibiotic administration, and the appropriateness of empirical therapy were not consistently reported across studies, which could have influenced the outcomes [61]. Future research should focus on conducting large-scale, high-quality randomized controlled trials to definitively determine the role and the true outcome of colistin combination therapy. Elements requiring particular attention include the patient’s sample size, characteristics of patient groups, PK/PD relationships and dosing, MIC determinations [62].

In addition to in vitro observations, several preclinical studies using animal models have explored the potential of colistin-based combinations against resistant pathogens. Another important aspect of combination therapy is its potential activity against pathogens expressing the *mcr-1* gene. Many studies have focused on combating chromosomally mediated colistin resistance, but the unique plasmid-mediated single gene resistance mechanism of *mcr-1* fundamentally distinguishes this resistance mechanism. Consequently, data derived from studies on chromosomal colistin resistance may not reliably predict the susceptibility of *mcr-1*–mediated resistance to antibiotic potentiation in combination with colistin [63]. MacNair et al. reported showing that *mcr-1*–expressing *Enterobacteriaceae* can be rendered susceptible to a broad spectrum of antibiotics when co-administered with colistin. Notably, this resensitization is achieved at sub-breakpoint concentrations of both colistin and the companion antibiotic, translating into therapeutic efficacy in murine infection models of the *mcr-1*–positive *Klebsiella pneumoniae* [63]. In a recent study, researchers indicated that the biotin biosynthesis inhibitor MAC13772 synergizes with colistin specifically against colistin-resistant bacteria by indirectly disrupting fatty acid synthesis (FAS) and restoring susceptibility to this last-resort antibiotic. Furthermore, they demonstrated that combinations of colistin with other FAS inhibitors, including cerulenin, triclosan, and Debio1452-NH3, exhibited broad activity against both chromosomal and plasmid-mediated colistin resistance. In addition, combination therapy with colistin and the clinically relevant FabI inhibitor Debio1452-NH3 was shown to be effective in murine models of systemic infection caused by *mcr-1*–positive *Klebsiella pneumoniae* and colistin-resistant *Escherichia coli* [64].

Overall, despite promising in vitro findings, the clinical benefit of colistin-based combination therapy remains uncertain and requires confirmation in well-designed randomized controlled trials. This discrepancy between mechanistic evidence derived from in vitro and preclinical studies and the inconsistent outcomes observed in clinical settings represents a major limitation in the current understanding of colistin combination therapy.

**Table 3:** Representative studies on colistin-based combination therapy. Positive outcomes of combination therapy are marked with a green arrow, whereas red arrows indicate no difference compared with monotherapy.

Combination	Pathogen	No. of Patients		Outcomes	Reference
colistin/meropenem	carbapenem-resistant *Acinetobacter baumannii*	71	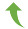	improves survival in critically ill patients	[65]
colistin/meropenem	carbapenem-resistant *Klebsiella pneumoniae*	60	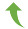	significant decrease in mortality not associated with any significant nephrotoxicity, hepatotoxicity, or neurotoxicity	[66]
colistin/fosfomycin	carbapenem-resistant *Acinetobacter baumannii*	94	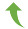	significantly more favorable microbiological response a trend toward more favorable clinical outcomes and lower mortality	[67]
colistin/meropenem	carbapenem-resistant *Acinetobacter baumannii*	324	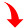	no significant differences in effectiveness and nephrotoxicity	[68]
tigecycline/colistin	carbapenem-resistant *Acinetobacter baumannii*	118	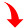	therapy with high dose colistin and standard dose tigecycline was not associated with lower crude mortality of bacteremia in critically ill patients	[69]
colistin/rifampicin	carbapenem-resistant *Acinetobacter baumannii*	43	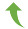 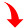	time to microbiological clearance was significantly shorter response rates were better, but differences were not significant	[70]
colistin/ampicillin-sulbactam	carbapenem-resistant *Acinetobacter baumannii*	39	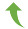	colistin and a high dose of ampicillin/sulbactam was associated with a more favorable clinical response to ventilator-associated pneumonia	[71]
colistin/carbapenem	carbapenem-resistant *Acinetobacter baumannii*	160	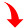	no significant difference in 14-day mortality no significant difference in clinical improvement and sputum-negative conversion	[72]
colistin/other antimicrobial agents	carbapenem-resistant Gram-negative bacteria	84	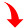	no significant differences regarding microbiologic and clinical outcomes	[73]
colistin/meropenem	carbapenem-resistant *Enterobacteriaceae*	406	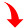	no significant difference between therapy	[74]
colistin/carbapenem	carbapenem-resistant *Klebsiella pneumoniae*	50	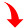	any noticeable advantage in terms of clinical response, microbiological response, nephrotoxicity, length of hospitalization, and mortality	[75]
tigecycline/colistin	carbapenem-resistant *Acinetobacter baumannii*	28	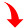	no significant differences in the 14-day, 30-day, in-hospital mortality rates, the rate of breakthrough bacteremia and the rate of bacterial eradication	[76]
colistin/rifampicin	carbapenem-resistant *Acinetobacter baumannii*	209	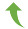	a significant increase in microbiologic eradication rate no difference for infection-related death and length of hospitalization	[77]
colistin/sitafloxacin	carbapenem-resistant *Acinetobacter baumannii*	56	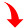	no significant difference in 28-day mortality no significant difference in adverse events	[78]
colistin/meropenem	carbapenem-resistant Gram-negative bacteria	423	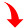	no difference in mortality, clinical failure, microbiologic cure or adverse events and neurotoxicity	[79]
intravenous colistin sulfate/other antimicrobial agents	carbapenem-resistant Gram-negative bacteria	80	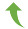 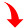	significantly higher clinical efficacy no significant difference in the 28-day mortality and length of hospital stay no significant difference in the incidence of adverse events	[80]
colistin/fosfomycin	carbapenem-resistant *Enterobacteriaceae*	220	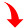 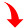 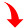	no significant difference in 30-day mortality no significant difference in the mortality at the end of treatment no significant difference in microbiologic response	[81]

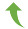
 positive outcome of combination therapy; 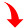 no significant difference in outcome of combination therapy.

## 6. Colistin Animal Usage

**Figure 3:**
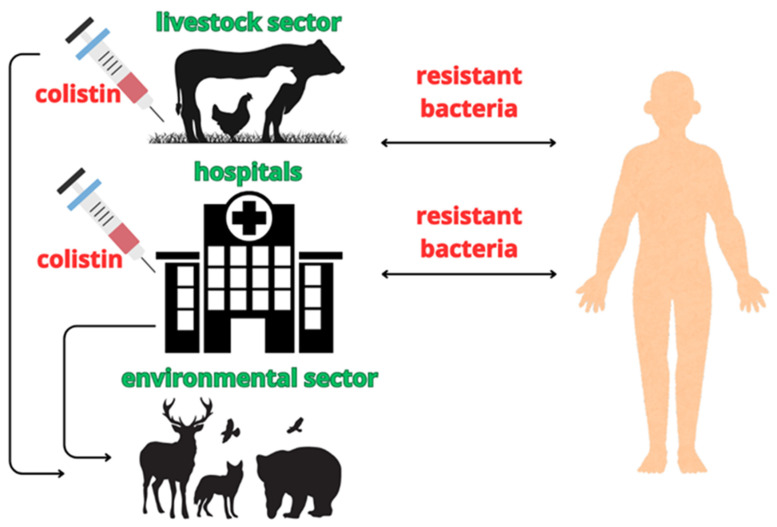
Possible transmission routes of colistin-resistant bacteria. Emergence of colistin-resistant strains as a result of the extensive use of colistin in the livestock sector and hospitals. Figure created using Canva (Canva Pty Ltd., Sydney, Australia).

Colistin has historically been widely used in food-producing animals across European countries, not only for the treatment of infections but also for prophylactic and metaphylactic purposes. This antibiotic is administered to a wide range of animals, including pigs, poultry, laying hens, and rabbits, as well as to milk-producing species such as cattle, sheep, and goats. The widespread use of colistin has contributed to the emergence and dissemination of antimicrobial-resistant (AMR) pathogenic and commensal bacteria within the intestinal tract of food-producing animals (Figure 3). These resistant bacteria may subsequently colonize the human microbiota through the food chain, either via handling or consumption of contaminated food products [82]. The latest data comparing the consumption of colistin among 28 EU/EEA member states show that the population-weighted mean of consumption is 340 times higher in food-producing animals compared to human medicine, according to the European Centre for Disease Prevention and Control (ECDC), the European Food Safety Authority (EFSA, 2017) [83], and the European Medicines Agency [84]. In several non-EU countries, colistin veterinary application also includes the treatment of salmonellosis and colibacillosis in livestock. Additionally, colistin is used to manage ear and eye infections in companion animals [85].

The use of polymyxins varies substantially between countries. In addition, no correlation was observed between polymyxin consumption in food-producing animals and that in humans within individual countries. In 2014, Italy, Spain, and Portugal reported the highest levels of polymyxin use in food-producing animals, whereas Greece, Ireland, and the United Kingdom demonstrated the greatest polymyxin consumption in human medicine after adjustment for biomass [82]. To preserve colistin as a last-resort antibiotic in human medicine, efforts have been made to restrict the use of polymyxins in food-producing animals. In 2016, EMA recommended that European Union member states should reduce the consumption of colistin in livestock below 5 mg/PCU (Population Correction Unit) by 2020 [84]. The latest data from EMA on polymyxin consumption demonstrate that six countries still exceeded this threshold in 2018, including Cyprus, Germany, Hungary, Poland, Portugal, and Romania [86]. Unfortunately, at present, a complete restriction of colistin use in livestock production is impossible due to its significance for the treatment of intestinal infections in pigs, poultry, and veal calves caused by *Salmonella* spp. or *E. coli* [82].

A major concern is that the use of colistin exerts strong selective pressure on bacterial populations in animals, promoting the co-selection of resistance to other classes of antibiotics. Colistin resistance determinants have been identified on a variety of mobilizable plasmids, including IncI2, IncX4, and IncFIA replicon types, which frequently co-harbor *mcr* genes alongside multiple antimicrobial resistance determinants. These plasmids often carry genes conferring resistance to several antibiotic classes, such as β-lactams (including penicillins and carbapenems), fluoroquinolones, aminoglycosides, tetracyclines, and trimethoprim–sulfonamide combinations [87]. Numerous studies indicate that the use of colistin in agriculture must be carefully considered, as increased mass medication practices are associated with a higher prevalence of colistin-resistant and multidrug-resistant bacteria, posing a significant threat to public health. Alternative preventive strategies, including vaccination, may help reduce morbidity and mortality from infectious diseases in food-producing animals [85]. From a One Health perspective, the continued use of colistin in livestock represents a significant concern, as agricultural reservoirs of colistin-resistant bacteria may contribute to the dissemination of resistance determinants across environmental, animal, and human microbial ecosystems.

## 7. Conclusions

Despite decades of clinical use, colistin remains a challenging antimicrobial agent in modern infectious disease therapy. Its reintroduction into clinical practice as a last-resort treatment for infections caused by multidrug-resistant Gram-negative bacteria has highlighted several unresolved issues. Susceptibility testing for colistin remains technically challenging. Several methodological limitations may compromise the accuracy of results, including adsorption of the drug to plastic laboratory materials, limited diffusion in agar, and the presence of heteroresistant bacterial subpopulations. Consequently, current recommendations from the European Committee on Antimicrobial Susceptibility Testing (EUCAST) designate broth microdilution in Mueller–Hinton broth as the reference method for reliable determination of colistin susceptibility. Nevertheless, the clinical use of colistin remains complicated due to its narrow therapeutic window. Achieving effective antibacterial activity requires plasma concentrations close to the toxicity threshold, which necessitates careful dose optimization, pharmacokinetic/pharmacodynamic (PK/PD) modeling, and therapeutic drug monitoring to balance efficacy with the risk of nephrotoxicity. Although combination regimens involving colistin have been widely proposed to enhance antibacterial efficacy and reduce the emergence of resistance, current evidence remains inconsistent. While numerous in vitro studies demonstrate synergistic interactions with other antimicrobials, these findings do not consistently translate into improved clinical outcomes in patients.

The global dissemination of plasmid-mediated resistance determinants, particularly *mcr* genes, represents a major threat to the long-term effectiveness of colistin. The presence of these genes on mobile plasmids facilitates their rapid spread across bacterial species and ecological niches. Given the clinical importance of colistin as a last-resort treatment for infections caused by multidrug-resistant Gram-negative bacteria, its use should be carefully controlled in both human and veterinary medicine. Continued surveillance and improved diagnostic methods are essential to preserve the efficacy of this critical antibiotic.

Taken together, the available evidence suggests that although colistin retains clinical relevance as a last-resort antibiotic, its therapeutic utility is constrained by significant pharmacological limitations, challenges in susceptibility testing, and a lack of consistent clinical benefit from combination therapies.

## 8. Graphical Preparation

Graphical elements and schematic illustrations included in the manuscript were prepared using Canva [88] and BioRender [89]. These platforms were used to design visual summaries and schematic representations of the analyzed information.

## Figures and Tables

**Table 1:** Comparison of the two colistin formulations currently available.

Feature	Colistin Sulfate	Colistin Methanesulfonate (Colistimethate Sodium)—CMS
Form	active	prodrug
MIC testing	recommended	not recommended
Elimination	non-renal	renal
Administration route	oral and topical, inhalation	parenteral, inhalation

## Data Availability

No new data were created or analyzed in this study. Data sharing is not applicable to this article.
